# Management of refractory macular hole with blood and gas-assisted autologous neurosensory retinal free flap transplantation: a case report

**DOI:** 10.1186/s12886-018-0909-9

**Published:** 2018-09-03

**Authors:** Pei-Kang Liu, Yo-Chen Chang, Wen-Chuan Wu

**Affiliations:** 1 Department of Ophthalmology, Kaohsiung Medical University Hospital, Kaohsiung Medical University, No. 100, Tzyou 1st Road, Kaohsiung, 807 Taiwan; 2 Department of Ophthalmology, Yuan’s General Hospital, No. 162 Cheng Kung 1st Road, Kaohsiung, 80249 Taiwan; 3 Institute of Biomedical Sciences, National Sun Yat-Sen University, 70 Lienhai Rd, Kaohsiung, 80424 Taiwan; 4 Department of Ophthalmology, School of Medicine, Kaohsiung Medical University, 100, Shih-Chuan 1st Road, Kaohsiung, 80708 Taiwan

**Keywords:** Autologous, Blood, Gas, Neurosensory, Free retinal flap, Transplantation, Refractory, Macular hole

## Abstract

**Background:**

Macular hole (MH) may become refractory if the hole does not close after multiple surgeries. We provide a modified surgical technique for refractory MH repair with neurosensory retinal free flap transplantation.

**Case presentation:**

To treat a 68-year-old female patient with refractory MH after multiple surgeries, we harvested a neurosensory retinal free flap with a 2-MH diameter area. A drop of whole blood was placed within the MH as an adhesive to fix the neurosensory retinal free flap at the MH under gas tamponade. Two months after surgery, optical coherence tomography (OCT) revealed closure of the MH. The flap was visible on OCT and had filled the MH without overlapping the neurosensory retina. The patient’s best-corrected visual acuity (BCVA) improved from 20/500 preoperatively to 20/50 at 2 months postoperatively.

**Conclusions:**

Using whole blood as an adhesive to aid in the fixation of an autologous neurosensory retinal free flap under gas tamponade provides another option for patients with refractory MH due to multiple prior surgeries.

**Electronic supplementary material:**

The online version of this article (10.1186/s12886-018-0909-9) contains supplementary material, which is available to authorized users.

## Background

With improvements in the surgical techniques for treating macular hole (MH), the success rate of MH repair is currently greater than 90% [1, 2]. However, in some cases, the MH still fails to close after repeated surgery, especially in patients with high levels of myopia [3] or chronic MH [4]. Several surgical techniques have been reported for treating refractory MH, such as extended internal limiting membrane (ILM) peeling, autologous free ILM flap transplantation, and lens capsular flap transplantation, [5] with or without an adjuvant blood component [6].

Grewal and Mahmoud reported a novel method for refractory myopic MH repair, which involved an autologous neurosensory retinal free flap of 2 disc diameters, perfluoro-n-octane heavy liquid (PFC; Perfluoron, Alcon) instillation, and direct PFC-silicone oil exchange [7]. In this technique, the surgeon needs to use both PFC and silicone oil to stabilize the free retinal flap. A small amount of PFC may remain in the eye, and the removal of silicone oil may be inevitable in the future. Flap dislocation after silicone oil removal is also possible. In addition, the relatively large size (2 disc diameters) of the retinal free flap may result in neurosensory retinal overlap.

To improve the surgical outcome and simplify the technique, we describe a newly modified technique involving the use of blood as an adhesive to aid in the fixation of the retinal free flap under gas tamponade with 15% perfluoropropane (C_3_F_8_).

## Case presentation

A 68-year-old female underwent phacoemulsification + intraocular lens implantation + pars plana vitrectomy (PPV) + ILM peeling + 18% sulfur hexafluoride (SF_6_) tamponade in January 2016 due to an epiretinal membrane and a lamellar MH. Unfortunately, macular hole retinal detachment (MHRD) occurred one month after surgery. She received PPV + extended ILM peeling + silicone oil tamponade in February 2016 and underwent removal of silicone oil in October 2016. The retina had attached well, although the MH became refractory, and her best-corrected visual acuity (BCVA) was 20/500. She underwent two PPV + free ILM flap transplantation + 15% C_3_F_8_ treatments in April 2017 and July 2017, with unsatisfactory results. Due to her repeated surgeries, an autologous free ILM flap could not be harvested. We decided to perform a neurosensory retinal free flap transplantation for the repair of this refractory MH after discussion with the patient.

**Fig. 1:**
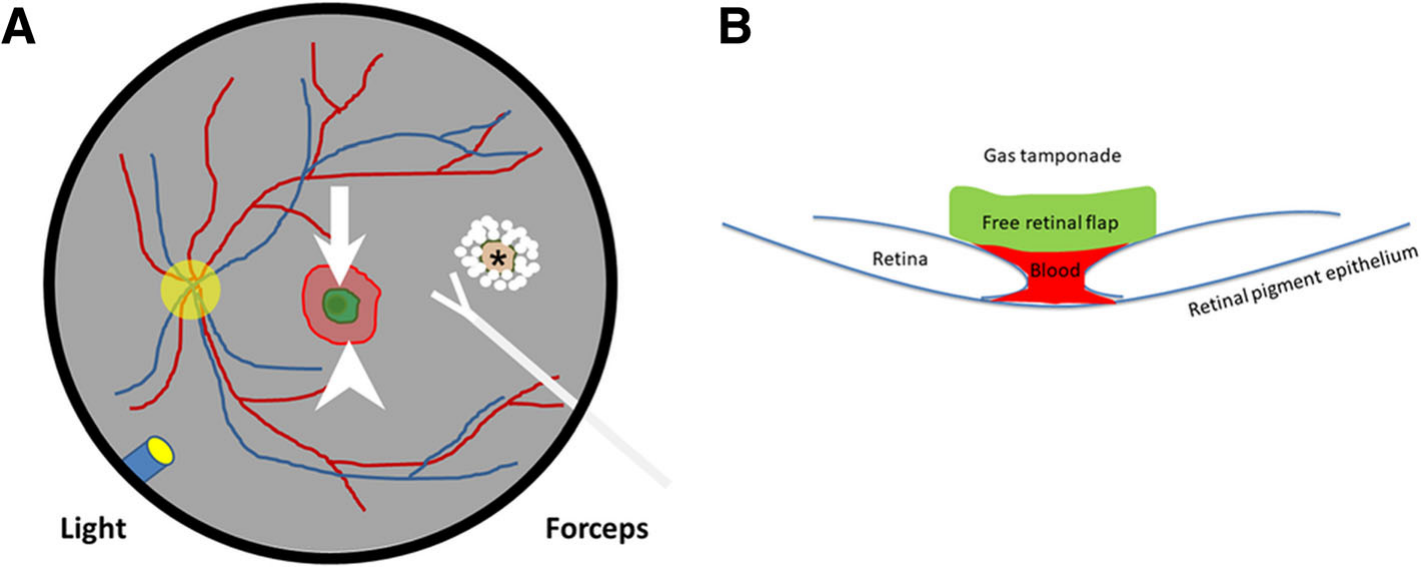
Schematic drawing of a surgical technique for the treatment of refractory MH involving autologous transplantation of a neurosensory retinal free flap using blood as an adhesive under gas tamponade. **a**. A neurosensory retinal free flap (white arrow) was harvested from the temporal side of the macula (asterisk) and was placed over the blood (white arrowhead) at the MH. **b**. Cross-sectional view of the neurosensory retinal free flap adhered to the blood in the MH area. MH, macular hole

A standard 25-g, 3-port PPV (Constellation; Alcon) was performed under general anesthesia. Endolaser photocoagulation was applied to outline the retinal free flap at the temporal retina. The neurosensory retinal free flap was approximately twice the diameter of the MH. The retina was cut with vertical scissors along the inner edge of the laser spots and was gently dissected with back-flush needle irrigation until a neurosensory retinal free flap with a 2-MH diameter area was harvested. The infusion was stopped temporarily to prevent turbulent flow. A drop of whole blood was placed within the MH, and the neurosensory retinal free flap was then placed on the blood. We performed fluid-gas exchange and flushed the vitreous cavity with 15% C_3_F_8_ at the end of the surgery (Fig. 1). All of the techniques were performed under standard 25-g, 3-port PPV. We did not use a bimanual approach under chandelier illumination (see Additional file 1). The patient was instructed to maintain a prone position for 14 days postoperatively and to avoid any unnecessary movement.

Three weeks after surgery, optical coherence tomography (OCT) revealed closure of the MH. The flap was visible on OCT and had filled the MH without overlapping of the neurosensory retina. The 2-month postoperative OCT examination still showed the MH closure. The patient reported an improvement of visual acuity and a decrease in her scotoma area. The patient’s BCVA improved from 20/500 preoperatively to 20/50 at 2 months postoperatively.

## Discussion

Refractory MH remains challenging for vitreoretinal surgeons and may progress to MHRD, which is a vision-threatening complication. Several methods, including extended ILM peeling, autologous free ILM flap transplantation, lens capsular flap transplantation, subretinal balanced salt solution injection to create macular detachment, and revitrectomy with autologous platelet concentrate (APC)/whole blood and gas tamponade, have been reported for refractory MH repair [5, 8, 9]. All these methods facilitate the closure of most MHs. However, a few patients still have a persistent MH even after multiple surgeries, and a free ILM flap may not be available. Furthermore, a lens capsular flap cannot be harvested in pseudophakic patients. Therefore, neurosensory retinal free flap transplantation becomes a reasonable and feasible method for the repair of a refractory MH.

Grewal and Mahmoud reported a novel method for the treatment of refractory MH with an autologous neurosensory retinal free flap with a 2-disc diameter under PFC assistance and silicone oil tamponade [7]. Parolini et al. then introduced another method involving an autologous choroid-retinal pigment epithelium-neurosensory retinal graft to treat end-stage exudative age-related macular degeneration [10]. Both methods provide potential surgical alternatives for the treatment of refractory MH. In the technique, the surgeons use both PFC and silicone oil to stabilize the retinal free flap. However, the stability of the transplanted retinal flap after removal of the silicone oil is unknown. Recurrent neurosensory retinal flap dislocation may occur.

**Fig. 2:**
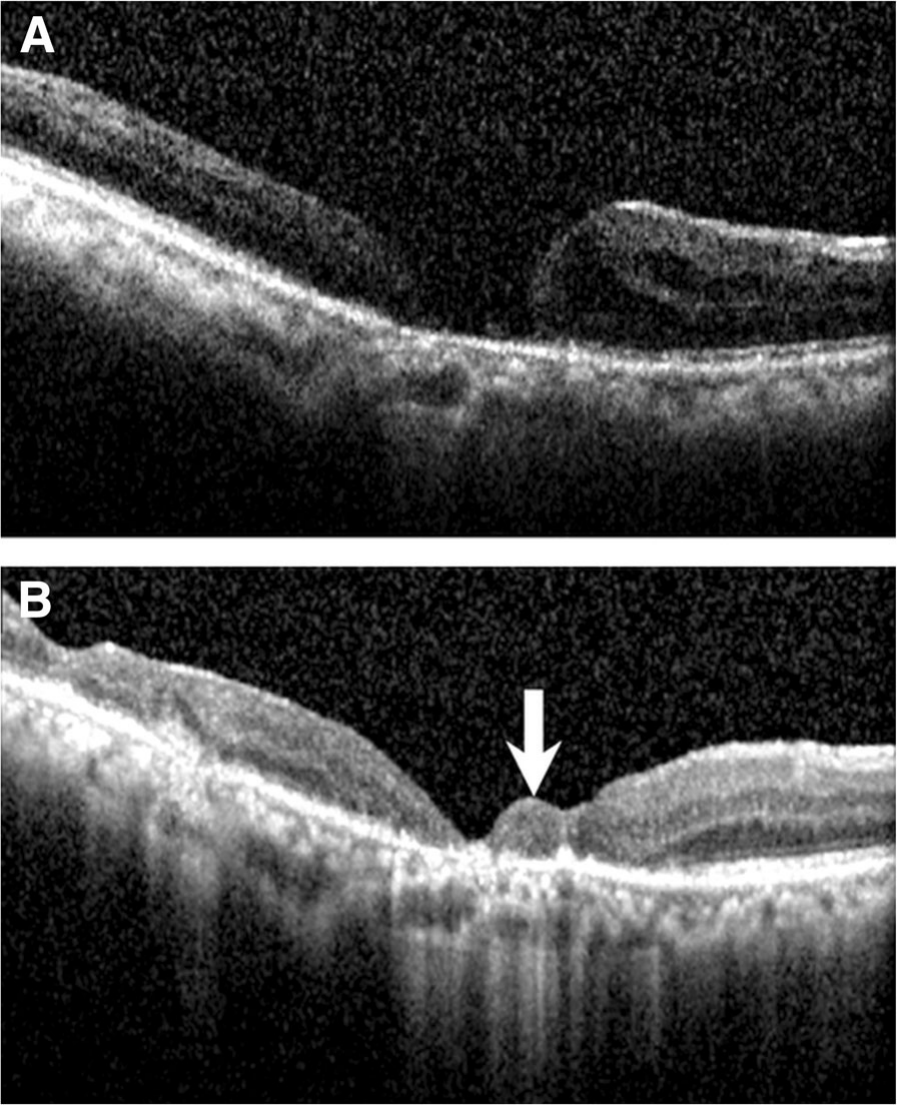
Serial OCT images. **a**. Preoperative OCT showing a chronic MH. **b**. Postoperative OCT showing closure of the MH with the neurosensory retinal free flap (white arrow) inside the MH. The patient’s BCVA improved from 20/500 preoperatively to 20/50 at 2 months postoperatively. OCT, optical coherence tomography; MH, macular hole; BCVA, best-corrected visual acuity

Purtskhvanidze et al. reported the use of APC or whole blood with gas tamponade for refractory MH repair [8]. In their report, revitrectomy with whole blood and gas achieved a lower closure rate than that with APC and gas (7.1% versus 85.2%, respectively). In addition, the retinal free flap was noted on OCT (Fig. 2b). Therefore, we speculate that closure of the refractory MH in this case was not from the effect of whole blood.

Whole blood contains serum, coagulants, albumin, and globulin and can be prepared as blood glue [11]. Therefore, it is reasonable to use whole blood as an adhesive to fix a neurosensory retinal free flap at a MH under gas tamponade. However, the relatively large size (2 disc diameters) of the retinal free flap may result in neurosensory retinal overlap. Therefore, we modified the size to 2 MH diameters to prevent neurosensory retinal overlap. Similar to the case reported by Grewal and Mahmoud, the restoration of retinal stratification with a corresponding increase in BCVA suggests that the autologous neurosensory retinal free flap may partially maintain retinal function in addition to acting as a scaffold for glial cell proliferation.

There are still several limitations to this technique. First, the most appropriate size of the neurosensory retinal free flap remains unclear since the free flap may contract after implantation into the MH. Second, due to the limited number of cases and relatively short follow-up times, the long-term outcomes and possible complications are unknown. We would like to emphasize that harvesting a neurosensory retinal free flap for transplantation remains the last resort in difficult cases to repair refractory MHs that fail to close after repeated surgeries. Third, a better adhesive or tissue glue for the fixation of the retinal free flap may improve the outcome. Additional experience may help refine the technique and clarify the anatomical and functional results.

## Conclusion

In conclusion, using whole blood as an adhesive to aid in the fixation of an autologous neurosensory retinal free flap under gas tamponade provides another choice for patients who are refractory to treatment due to prior multiple surgeries and may decrease the use of PFC and silicone oil, although a proper trial or further studies are necessary to confirm the effect. We present this modified technique with a lower learning curve in an attempt to simplify the surgical procedure. The method seems to be suitable, reasonable, and affordable for every patient. We hope to provide another option to all vitreoretinal surgeons worldwide for refractory or chronic MH repair.

## Additional file

Additional file 1: Surgical video. (MP4 70714 kb)

## Abbreviations

APC: autologous platelet concentrate
BCVA: Best-corrected visual acuity
C_3_F_8_: Perfluoropropane
ILM: Internal limiting membrane
MH: Macular hole
MHRD: Macular hole retinal detachment
OCT: Optical coherence tomography
PFC: Perfluoro-n-octane heavy liquid
PPV: Pars plana vitrectomy
SF_6_: Sulfur hexafluoride
